# Healthcare Service Disparities in Cancer Rehabilitation and Treatment Costs in Japan: A Cross-Sectional Analysis of National Data

**DOI:** 10.7759/cureus.80100

**Published:** 2025-03-05

**Authors:** Hirotomo Shibahashi, Kanta Ohno, Shinpei Ikeda, Yosuke Seike

**Affiliations:** 1 Occupational Therapy, Department of Rehabilitation, School of Health Sciences, Tokyo University of Technology, Tokyo, JPN

**Keywords:** cancer in japan, cancer rehabilitation, cancer treatment costs, healthcare provision, oncology, regional disparities

## Abstract

Background: Cancer rehabilitation is essential for addressing the physical, psychological, and social challenges associated with cancer treatment. It plays a crucial role in mitigating functional impairment and enhancing recovery. However, the patterns of its utilization in relation to different cancer treatment modalities remain insufficiently understood. This study aimed to analyze the utilization of cancer rehabilitation in Japan and examine its association with surgery, chemotherapy, and radiation therapy.

Methods: A cross-sectional study was conducted using standardized claims data ratio (SCR) scores from Japan's National Database (NDB) between 2017 and 2021. SCRs for surgery, chemotherapy, radiotherapy, and cancer rehabilitation costs were analyzed across regions. Statistical analyses included repeated-measures analysis of variance (ANOVA) and a mixed-effects model to assess the influence of treatment type on rehabilitation costs.

Results: Surgery showed significant year-to-year differences (*p* < 0.05), whereas rehabilitation, radiotherapy, and chemotherapy did not. Radiotherapy (*p* = 0.03) and chemotherapy (*p* = 0.01) increased rehabilitation costs, whereas the interactions between surgery and chemotherapy (*p* = 0.03) and between chemotherapy and radiotherapy (*p* = 0.01) significantly reduced costs, suggesting the potential cost-mitigating potential of multimodal treatment strategies.

Conclusions: This study demonstrated that although individual cancer treatments increase rehabilitation costs, treatment interactions can help mitigate these financial burdens, highlighting the importance of integrated care. Our findings support the incorporation of rehabilitation, including multidisciplinary approaches, into cancer survivorship care. Further research is needed to optimize rehabilitation planning based on treatment interactions to improve cost-efficiency and clinical outcomes.

## Introduction

Cancer rehabilitation is an integral component of comprehensive cancer care, aimed at mitigating the physical, psychological, and social challenges patients encounter throughout their disease trajectory [1-5]. In Japan, cancer remains the leading cause of mortality, accounting for 24.3% of all deaths in 2023 [6]. With advancements in oncological treatment modalities [7-9], many patients seek to restore function and enhance their quality of life before and after surgery, radiation therapy, and chemotherapy [7]. Moreover, as Japan's population continues to age, the incidence of cancer is expected to rise significantly, necessitating the expansion of effective rehabilitation programs to meet the growing needs of patients [2,10].

Cancer treatment often involves multiple complex modalities. Surgical interventions, particularly for breast, head, and neck cancers [11-14], often result in long-term impairment of mobility and function, thereby limiting patients’ ability to regain independence. Furthermore, radiation therapy and chemotherapy frequently lead to debilitating side effects such as fatigue and neuropathy, which significantly hinder post-treatment recovery and daily activities [15,16]. Evidence indicates that structured rehabilitation programs play a crucial role in addressing these functional impairments, accelerating recovery, and improving overall physical and psychological well-being [4,5,17-21].

Recently, the increasing availability of open healthcare data has provided unprecedented opportunities to systematically assess the effectiveness and utilization of rehabilitation services on a national scale [22]. Although the benefits of cancer rehabilitation are well documented [21,22], including improvements in insomnia, dyspnea, and quality of life, a comprehensive understanding of how different cancer treatment modalities influence rehabilitation utilization remains limited.

Study objectives

This study aimed to investigate the patterns of cancer rehabilitation utilization in Japan using publicly available National Health Insurance claims data [23]. Specifically, we examined annual trends in surgery, chemotherapy, radiation therapy, and rehabilitation and analyzed the association between rehabilitation utilization and these treatment modalities. Furthermore, we evaluated whether multimodal treatment strategies influence rehabilitation expenditures compared to monotherapy.

By clarifying these trends, this study provides critical insights for policy interventions, optimizes rehabilitation service allocation, and promotes the integration of rehabilitation into standard cancer care, ultimately improving patient outcomes.

## Materials and methods

Data sources

This study used standardized claims data ratio (SCR) scores, which are sex- and age-adjusted indices derived from the National Database (NDB) open data, made publicly available by the Cabinet Office. The NDB of Health Insurance Claims and Specific Health Checkups of Japan [24], administered by the Ministry of Health, Labor and Welfare, encompasses a comprehensive collection of administrative claims data for nearly all healthcare services delivered in Japan, providing an extensive resource for analyzing healthcare utilization patterns nationwide.

SCR scores were calculated using NDB data and were designed to standardize healthcare service utilization across regions by adjusting for differences in demographic structures (sex and age composition). SCR represents the ratio of observed healthcare service counts to expected healthcare service counts, expressed as

\( SCR = \frac{\sum \textit{Observed claims by sex and age group}}{\sum \textit{Expected claims by sex and age group}} \times 100 \)

Since SCR is calculated based on NDB data, the exact number of individual cases is not available. However, we analyzed SCR data from all 47 prefectures in Japan for 2017-2021.

The expected claims were calculated to be: $\sum (\textit{Population of each region by sex and age group}) \times(\textit{National claims rate by sex and age group})$

In this formula, the observed claims represent the actual number of healthcare service claims recorded within a specific region, categorized by sex and age. The expected claims were calculated by multiplying the population of each region (stratified by sex and age group) by the corresponding national claims rate for the same sex and age group.

An SCR score of 100 indicates that healthcare service provision in the region matches the national average after adjusting for sex and age. A score above 100 suggests higher-than-expected utilization relative to the adjusted population size, whereas a score below 100 indicates lower-than-expected utilization.

This study used publicly available data that were fully anonymized and contained no personally identifiable information. The data were originally collected and published by the government as official statistics for policy purposes, ensuring that they were anonymized and could not be used to identify individuals. No personal data were collected.

No variables were excluded due to missing data. While some missing values were present in the surgical procedure data (ranging from 1.10% to 13.48% across years), there were no missing values for radiotherapy, chemotherapy, or rehabilitation utilization. The overall missing data rate across the dataset was approximately 10%, with all years having less than 15% missing data. Given this moderate level of missingness, we proceeded with the analysis without performing imputation.

Ethical concerns

This study utilized publicly available, de-identified data, and therefore, it was deemed exempt from ethical review by the Ethics Committee of Tokyo University of Technology. According to the institution’s policy, studies using publicly available and fully anonymized datasets do not require institutional review board approval.

Study design and population

In this cross-sectional study, conducted at Tokyo University of Technology, Japan, we utilized freely accessible SCR data to evaluate healthcare provision. The analysis began by accessing the Cabinet Office website and downloading prefecture-level files for medical service branch codes from 2017 to 2021 [23]. Data related to surgeries explicitly mentioning “malignant tumors” in the procedure name were extracted. The cancer categories included the skin, soft tissues of the limbs and trunk, pharynx, larynx, tongue, thyroid, breast, mediastinum, lung, esophagus, retroperitoneum, gallbladder, hilar bile duct, colon, large intestine, kidney, ureter, bladder, testis, prostate, uterus, and uterine appendages. Because data for 2017 were unavailable for chemotherapy and radiotherapy, data from 2018 to 2021 were used for lung cancer, metastatic lung cancer (only for 2021), gastric cancer, colorectal cancer, liver cancer, metastatic liver cancer (only for 2021), and breast cancer. Rehabilitation cost data for cancer patients from 2017 to 2021 were extracted. All data extracted in this study were limited to inpatient cases.

Statistical analysis

The mean SCRs for all surgeries, radiation therapies, chemotherapies, and rehabilitation costs of patients with cancer for all malignant tumors across each prefecture and each year were calculated. A repeated-measures analysis of variance (ANOVA) was subsequently conducted, and Bonferroni's multiple comparison test was used for post-hoc analysis. To evaluate the effects of surgery, radiation therapy, and chemotherapy on the rehabilitation costs of patients with cancer, a mixed-effects model analysis was performed. Rehabilitation costs of patients with cancer were set as the dependent variables, while surgery, radiation therapy, chemotherapy, and their interaction terms as fixed effects. To account for regional variations, we used a mixed-effects model in which each prefecture was treated as a random effect. This approach allowed us to model the hierarchical structure of the data by considering within-prefecture correlations and inter-regional variability. Thus, the analysis appropriately adjusts for potential region-specific effects that may influence healthcare utilization patterns. All analyses were conducted using R version 4.2.1 (R Foundation for Statistical Computing, Vienna, Austria) and EZR for Windows version 1.65 (Saitama Medical Center, Jichi Medical University) [25], with a significance level set at 5%.

## Results

**Figure 1:**
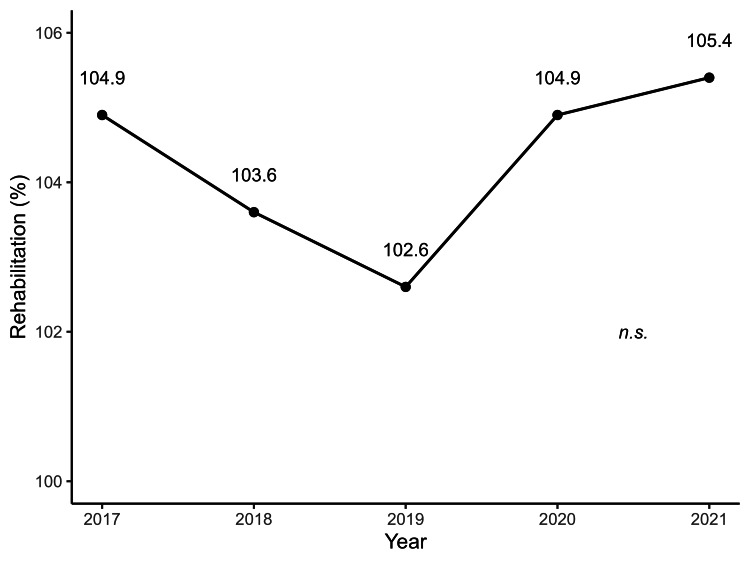
Annual trends in rehabilitation costs for patients with cancer from 2017 to 2021 Each point represents the mean rehabilitation cost per year, with a line illustrating the trend over time. "n.s." indicates no significant difference between years. Data are based on SCR calculations from 47 prefectures (2017–2021). SCR: standardized claim-data ratio

**Figure 2:**
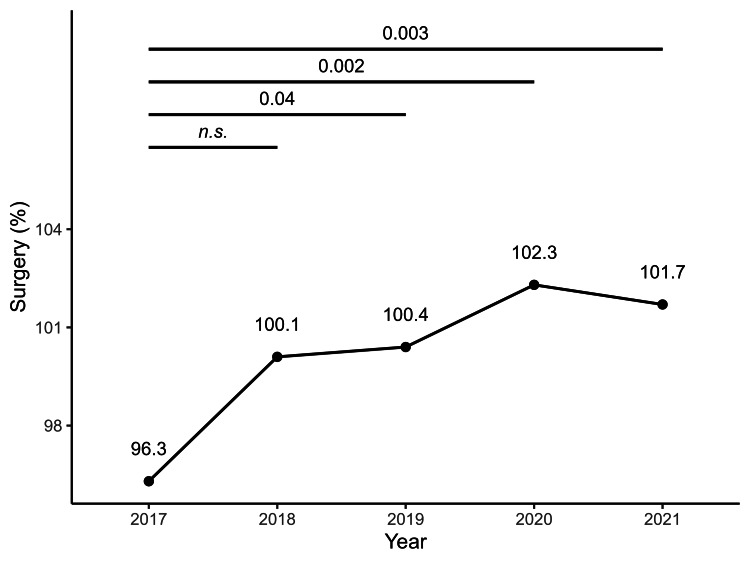
Annual trends in surgical costs for cancer treatment from 2017 to 2021 Each point shows the mean surgical cost per year, with a line indicating the trend. Data are based on SCR calculations from 47 prefectures (2017–2021). SCR: standardized claim-data ratio

**Figure 3:**
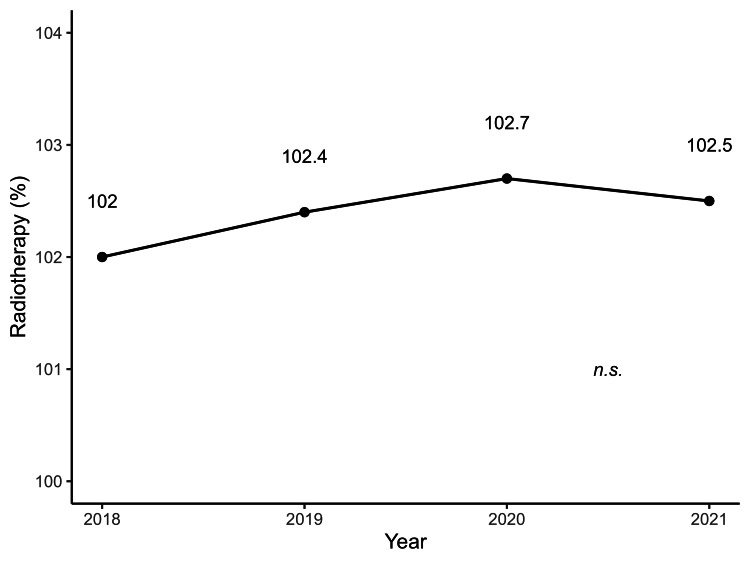
Annual trends in radiotherapy costs for patients with cancer from 2018 to 2021 Each point represents the mean radiotherapy cost per year, with a line illustrating the trend over time. No significant differences were observed between years. Data are based on SCR calculations from 47 prefectures (2017–2021). SCR: standardized claim-data ratio

**Figure 4:**
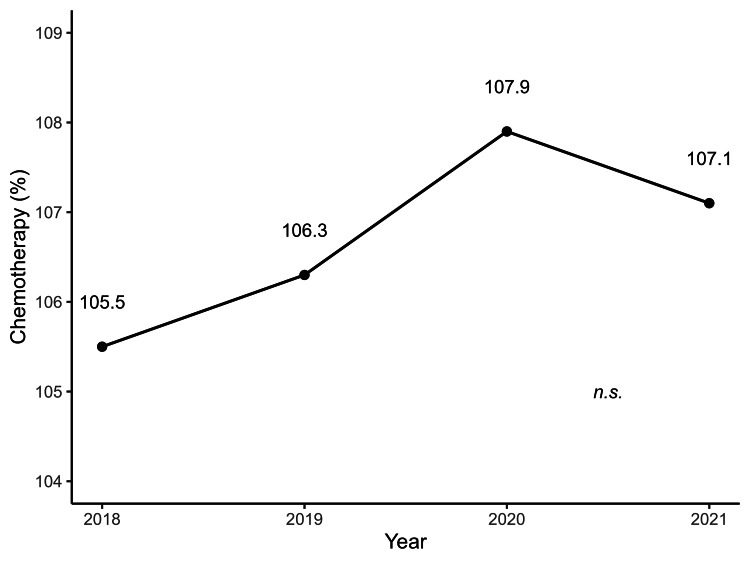
Annual trends in chemotherapy costs for patients with cancer from 2018 to 2021 Each point shows the mean chemotherapy cost per year, with a line indicating the trend. No significant differences were observed between years. Data are based on SCR calculations from 47 prefectures (2017–2021). SCR: standardized claim-data ratio

Figures 1-4 illustrate the annual trends in the mean SCR values for surgery, radiotherapy, chemotherapy, and cancer rehabilitation costs. In the post-hoc Bonferroni tests following repeated-measures ANOVA, significant differences in SCR values for surgery were observed when comparing 2017 values with that of 2019 (*p* = 0.04), 2020 (*p* = 0.002), and 2021 (*p* = 0.003), whereas no significant differences were observed for cancer rehabilitation, radiotherapy and chemotherapy. The year-by-year trends in SCR values for each type of surgery are shown in Table 1, with laparoscopic bladder and prostate cancer surgeries maintaining high SCR values since 2018. Tables 2-3 represent annual SCR trends by treatment site for radiotherapy and chemotherapy, respectively. Data for metastatic lung and liver cancers are available only for 2021, and the SCR values for breast cancer remained consistently high throughout the observation period.

**Table 1:** Annual mean SCR scores by surgical procedure for malignant tumors SCR: standardized claim-data ratio; N.A.: not applicable

Surgical procedure	2017, mean± SD	2018, mean± SD	2019, mean± SD	2020, mean± SD	2021, mean± SD
Excision of malignant skin tumor	102.0±28.2	100.4±27.7	101.8±28.2	102.3±25.7	102.1±27.5
Surgery for malignant soft tissue tumor of the extremities and trunk	92.9±45.8	93.0±39.3	96.3±42.9	97.5±38.0	95.0±42.0
Surgery for malignant pharyngeal tumor	88.0±54.9	99.5±48.0	92.2±53.2	111.3±58.8	110.3±46.8
Surgery for malignant laryngeal tumor	100.7±36.9	96.8±30.6	100.6±36.6	105.3±43.1	101.6±39.7
Surgery for malignant tongue tumor	93.2±37.7	99.4±38.3	93.4±35.7	98.8±40.3	98.0±39.5
Surgery for malignant thyroid tumor	100.7±46.8	99.9±44.5	98.8±44.9	100.2±36.9	99.8±41.4
Surgery for malignant breast tumor	92.5±13.3	94.6±10.6	94.3±10.8	95.2±10.5	95.7±10.1
Thoracoscopic surgery for malignant mediastinal tumor	N.A.	100.2±42.5	98.5±10.8	99.8±37.0	100.2±35.4
Surgery for malignant lung tumor	90.9±46.9	86.8±41.5	85.3±39.5	86.7±39.5	88.3±38.2
Thoracoscopic surgery for malignant lung tumor	101.1±23.1	101.6±22.5	101.0±21.8	101.0±21.3	100.2±18.5
Thoracoscopic surgery for malignant esophageal tumor	96.2±47.0	94.3±39.8	92.8±43.2	91.9±39.7	90.9±37.8
Surgery for malignant retroperitoneal tumor	89.6±37.7	95.6±34.7	95.8±34.8	94.8±31.1	92.5±36.1
Surgery for malignant gallbladder tumor	100.6±30.9	101.7±30.7	106.1±31.3	107.4±30.5	107.2±34.5
Surgery for malignant perihilar bile duct tumor	N.A.	N.A.	96.8±30.5	98.9±29.4	102.9±33.7
Laparoscopic excision of malignant colonic tumor	94.9±18.6	96.3±19.0	95.5±16.9	98.2±17.3	96.9±16.1
Endoscopic submucosal dissection for early malignant colorectal tumor	87.7±29.5	87.7±34.5	89.3±33.8	90.5±31.1	89.8±30.6
Surgery for malignant renal (ureteral) tumor	95.1±51.5	95.9±54.5	98.2±58.7	99.6±59.7	104.2±63.8
Laparoscopic surgery for malignant renal (ureteral) tumor	97.2±30.3	99.2±30.1	98.7±28.8	99.7±24.5	99.2±24.1
Laparoscopic surgery for malignant renal tumor	98.9±52.0	114.6±41.5	111.2±43.7	109.7±37.9	105.2±36.4
Surgery for malignant bladder tumor	96.9±15.4	97.0±14.9	96.8±14.9	97.6±14.7	97.1±14.0
Laparoscopic surgery for malignant bladder tumor	N.A.	137.2±73.9	126.0±51.5	122.1±48.9	112.9±41.0
Surgery for malignant testicular tumor	97.2±22.3	95.3±24.6	97.3±24.6	98.0±23.4	99.6±24.3
Surgery for malignant prostate tumor	107.1±85.9	117.6±95.1	127.2±109.6	142.0±111.6	153.0±139.3
Laparoscopic surgery for malignant prostate tumor	95.1±35.4	187.0±152.4	211.4±177.1	223.7±212.0	223.5±236.4
Surgery for malignant uterine tumor	98.1±17.3	98.8±19.4	98.5±19.6	99.0±17.1	98.6±22.3
Laparoscopic surgery for malignant uterine tumor	N.A.	115.3±47.4	112.3±47.5	103.7±43.2	95.1±34.5
Surgery for malignant tumor of the uterine adnexa	95.3±21.2	95.6±20.3	93.2±20.8	92.8±21.0	95.4±19.6

**Table 2:** Annual mean SCR scores by radiotherapy SCR: standardized claim-data ratio; N.A.: Not applicable

Radiotherapy	2018, mean ± SD	2019, mean ± SD	2020, mean ± SD	2021, mean ± SD
Lung cancer	101.2±23.5	101.8±23.9	101.7±22.8	100.4±23.3
Metastatic lung cancer	N.A.	N.A.	N.A.	103.0±36.7
Gastric cancer	100.7±34.4	102.1±37.9	103.4±39.8	99.0±34.8
Colorectal cancer	100.9±26.0	100.8±27.6	99.5±28.2	102.3±28.2
Hepatocellular carcinoma	99.9±27.2	101.8±31.9	101.9±31.9	107.2±50.4
Metastatic liver cancer	N.A.	N.A.	N.A.	102.7±40.8
Breast cancer	107.1±53.1	105.7±58.0	107.2±28.1	103.3±50.0

**Table 3:** Annual mean SCR scores by chemotherapy SCR: standardized claim-data ratio; N.A.: not applicable

Chemotherapy	2018, mean ± SD	2019, mean ± SD	2020, mean ± SD	2021, mean ± SD
Lung cancer	101.7±21.1	102.0±20.9	103.2±23.0	102.6±20.1
Metastatic lung cancer	N.A.	N.A.	N.A.	109.1±44.5
Gastric cancer	107.7±30.8	109.2±31.1	111.9±35.8	112.8±38.4
Colorectal cancer	102.8±39.8	103.7±39.9	106.0±42.6	108.3±44.2
Hepatocellular carcinoma	104.4±29.3	105.6±31.8	107.1±35.6	108.4±41.0
Metastatic liver cancer	N.A.	N.A.	N.A.	97.5±60.3
Breast cancer	111.0±44.7	110.9±46.5	111.2±47.2	111.2±48.8

A mixed-effects model was used to examine the impact of various cancer treatments (surgery, chemotherapy, and radiotherapy) on rehabilitation costs. The fixed effects included surgery, chemotherapy, radiotherapy, and interaction terms with random intercepts for the year to account for annual variability (Table 4).

**Table 4:** Mixed effects model results on cancer treatments affecting cancer patient rehabilitation costs Interaction terms (e.g., Surgery*Radiotherapy) indicate the combined effect of the two treatments. SCR: standardized claim-data ratio; CI: confidence interval

Variable	Estimate	Standard error	95% CI	p value
Surgery	3.97	2.36	-0.66－8.60	0.09
Radiotherapy	4.98	2.24	0.59－9.37	0.03
Chemotherapy	5.86	2.37	1.21－10.51	0.01
Surgery*Radiotherapy	-0.05	0.02	-0.09－-0.01	0.05
Surgery*Chemotherapy	-0.05	0.02	-0.09－-0.01	0.03
Radiotherapy*Chemotherapy	-0.06	0.02	-0.10－-0.02	0.01

Surgery had a positive, although non-significant, effect on cancer rehabilitation costs (estimate = 3.97, standard error (SE) = 2.36, 95%CI = -0.66, 8.60, *p* = 0.09). In contrast, chemotherapy was significantly associated with increased rehabilitation costs (estimate = 5.86, SE = 2.37, 95%CI = 1.21, 10.51, *p* = 0.01), as was radiotherapy (Estimate = 4.98, SE = 2.24, 95%CI = 0.59, 9.37, *p* = 0.03). As for the interaction effects, the combination of surgery and chemotherapy yielded a significant negative effect on rehabilitation costs (estimate = -0.05, SE = 0.02, 95%CI = -0.09, -0.01, *p* = 0.03). Similarly, the interaction between surgery and radiotherapy indicated a significant borderline reduction in costs (estimate = -0.05, SE = 0.02, 95%CI = -0.09, -0.01, *p* = 0.05). The interaction between chemotherapy and radiotherapy also showed a significant negative effect on rehabilitation costs (estimate = -0.06, SE = 0.02, 95%CI = -0.10, -0.02, *p* = 0.01).

## Discussion

This study analyzed publicly available SCR data to investigate annual trends in rehabilitation expenditures associated with surgery, chemotherapy, and radiotherapy in patients with cancer. Using a mixed-effects model, we examined how different treatment modalities, and their interactions influenced rehabilitation costs. The key findings indicate that monotherapy with surgery, chemotherapy, or radiotherapy was associated with increased rehabilitation costs, whereas the combination of these treatments significantly reduced expenses. Radiotherapy and chemotherapy independently led to a significant increase in rehabilitation costs (*p* = 0.03 and *p* = 0.01, respectively). This increase may be attributed to the higher frequency of rehabilitation sessions required for managing treatment-related complications such as fatigue, neuropathy, and musculoskeletal impairments, which are commonly observed in patients undergoing these therapies. Previous studies have reported that chemotherapy-induced peripheral neuropathy and radiation-related tissue damage can necessitate prolonged rehabilitation interventions to restore functional independence and quality of life [15-17]. Further research is needed to determine whether the observed cost increase is primarily driven by a greater number of rehabilitation sessions or extended rehabilitation duration. Conversely, the interaction effects between surgery and radiotherapy, as well as between chemotherapy and radiotherapy, demonstrated a cost-reducing impact (*p* = 0.03 and *p* = 0.01, respectively). Since SCR is a ratio-based metric, changes in SCR scores may be partially influenced by variations in total treatment expenditures. However, our findings also suggest that treatment interactions themselves may play a role in shaping rehabilitation needs. The observed decrease in standardized rehabilitation SCR scores for combination therapies does not necessarily imply reduced rehabilitation service utilization but rather reflects a complex interplay between treatment modalities that may influence recovery patterns and rehabilitation demands. Further research is needed to disentangle the effects of financial expenditures and clinical rehabilitation needs in driving these trends.

A previous study by Mewes et al. demonstrated that various cancer treatments, including surgery, chemotherapy, and radiotherapy, significantly contribute to rehabilitation expenditures, emphasizing the financial burden on patients [25]. However, they primarily focused on the direct impact of cancer treatment on rehabilitation costs. Our study expands this knowledge by examining the economic implications of treatment interactions, highlighting how different treatment modalities influence rehabilitation expenditures when combined.

Systematic reviews have further confirmed that cancer care imposes substantial out-of-pocket costs, including rehabilitation expenses, particularly for patients undergoing intensive treatment regimens [26]. Additionally, exercise-based rehabilitation interventions are cost-effective in cancer survivors, suggesting potential strategies to mitigate financial burdens associated with post-treatment rehabilitation [27]. However, these studies primarily examined the independent effects of specific treatment modalities, whereas our findings suggest that integrated treatment strategies may alleviate long-term rehabilitation costs. This represents a novel contribution to the literature by demonstrating that combining different cancer treatment modalities can optimize rehabilitation expenditures. The observed cost-reducing effects of integrated treatment strategies may be attributed to more efficient rehabilitation planning, reduced duplication of services, and improved coordination of care, ultimately leading to optimized resource utilization. These findings align with those of previous studies, suggesting that well-coordinated oncology care can minimize post-treatment complications and reduce overall healthcare expenditures [28,29]. However, further research is needed to examine how specific rehabilitation interventions can maximize these cost-saving benefits.

Given these findings, a multidisciplinary approach that integrates rehabilitation within standard oncology care could play a crucial role in improving both cost-efficiency and patient outcomes. In Japan, the Cancer Control Plan emphasizes the importance of rehabilitation; however, implementation challenges persist, such as reimbursement limitations and lack of standardized rehabilitation pathways [2]. Addressing these barriers through targeted policy revisions, such as expanding outpatient rehabilitation coverage and enhancing provider education, may facilitate a more effective integration of rehabilitation services into oncology care. Incorporating rehabilitation into standardized cancer treatment pathways may be a viable strategy for optimizing healthcare costs in Japan. Expanding insurance coverage for integrated rehabilitation programs and enhancing interdisciplinary collaboration may further facilitate the adoption of cost-effective rehabilitation strategies in Japan’s cancer control plans. Future studies should explore the feasibility of cost-effective rehabilitation models tailored to Japan’s healthcare system to ensure that cancer rehabilitation strategies align with global best practices while addressing country-specific infrastructure and resource constraints.

Limitations

Although this study provided valuable insights into the economic and functional aspects of cancer rehabilitation, it had certain limitations. First, our analysis relied on national databases and publicly available data, which may be subject to variability in data-recording practices across healthcare institutions. This variability could introduce inconsistencies, particularly in cost assessments across treatment modalities and geographic regions. Additionally, the dataset did not contain individual-level information, limiting our ability to adjust for potential confounding factors such as socioeconomic status, disease severity, or comorbidities. Although SCRs account for age and sex differences, they do not fully control for variations in healthcare access, institutional practices, or regional economic disparities, necessitating cautious interpretation of the findings when comparing different populations. Second, information regarding the specific cancer types for which rehabilitation services were provided was not publicly available, precluding stratified analyses based on cancer sites. Moreover, the dataset did not distinguish between curative and palliative radiation therapies. Given that curative radiation therapy aims to eradicate malignancies, whereas palliative radiation therapy primarily focuses on symptom relief and quality of life improvement, this distinction could have significant implications for rehabilitation needs. The inability to differentiate between these treatment interventions may have introduced heterogeneity into the analysis, potentially affecting the interpretation of the findings. 

Finally, the generalizability of our findings should be considered in the context of Japan’s healthcare system, which has unique reimbursement structures and accessibility factors. Differences in rehabilitation service coverage, treatment protocols, and healthcare policies must be acknowledged when applying these findings to other healthcare settings. Future studies should examine whether similar trends are observed in diverse healthcare systems with varying reimbursement models and cancer care frameworks. To address these limitations, future research should incorporate more granular individual-level data, including cancer-specific rehabilitation details and treatment intent classifications, to refine cost analyses and better elucidate the economic and clinical impacts of rehabilitation interventions. Additionally, prospective studies are needed to confirm the cost-saving mechanisms observed in this study, particularly in the context of treatment interactions, such as surgery and chemotherapy.

## Conclusions

This study demonstrates that although individual cancer treatment modalities substantially impact rehabilitation costs, interactions between treatment types can mitigate these financial burdens, underscoring the importance of integrated care strategies. Our findings align with existing evidence supporting the inclusion of rehabilitation interventions, including multidisciplinary approaches, as components of cancer survivorship care. However, further research is required to optimize rehabilitation planning based on treatment interactions to ensure cost-effectiveness and clinical effectiveness.

## References

[1] Fialka-Moser V, Crevenna R, Korpan M, Quittan M (2003). Cancer rehabilitation: particularly with aspects on physical impairments. J Rehabil Med.

[2] Fukushima T, Tsuji T, Watanabe N (2022). Cancer rehabilitation provided by designated cancer hospitals in Japan: the current state of outpatient setting and coordination after discharge. Prog Rehabil Med.

[3] Lange M, Joly F, Vardy J (2019). Cancer-related cognitive impairment: an update on state of the art, detection, and management strategies in cancer survivors. Ann Oncol.

[4] Christensen JF, Jones LW, Andersen JL, Daugaard G, Rorth M, Hojman P (2014). Muscle dysfunction in cancer patients. Ann Oncol.

[5] Silver JK, Baima J, Mayer RS (2013). Impairment-driven cancer rehabilitation: an essential component of quality care and survivorship. CA Cancer J Clin.

[6] (2025). Ministry of Health, Labour, and Welfare: 2023 demographic statistics monthly annual report (approximate). https://www.mhlw.go.jp/toukei/saikin/hw/jinkou/geppo/nengai23/.

[7] An KY, Min J, Lee DH, Kang DW, Courneya KS, Jeon JY (2024). Exercise across the phases of cancer survivorship: a narrative review. Yonsei Med J.

[8] Wyld L, Audisio RA, Poston GJ (2015). The evolution of cancer surgery and future perspectives. Nat Rev Clin Oncol.

[9] Verma A, Rawat A, Sahu V, Chaurasia Y, Kumar A, Rathore S, Richa Tripathi (2024). Current advances in cancer treatment:a comprehensive review of therapeutic strategies and emerging innovations. World J Bio Pharm Health Sci.

[10] Takahashi M (2023). Cancer survivorship care: challenges and opportunities in Japan. Jpn J Clin Oncol.

[11] Marco E, Trépanier G, Chang E, Mauti E, Jones JM, Zhong T (2023). Postmastectomy functional impairments. Curr Oncol Rep.

[12] Chrischilles EA, Riley D, Letuchy E (2019). Upper extremity disability and quality of life after breast cancer treatment in the Greater Plains Collaborative clinical research network. Breast Cancer Res Treat.

[13] Guru K, Manoor UK, Supe SS (2012). A comprehensive review of head and neck cancer rehabilitation: physical therapy perspectives. Indian J Palliat Care.

[14] Eickmeyer SM, Walczak CK, Myers KB, Lindstrom DR, Layde P, Campbell BH (2014). Quality of life, shoulder range of motion, and spinal accessory nerve status in 5-year survivors of head and neck cancer. PM R.

[15] Hsiao CP, Daly B, Saligan LN (2016). The etiology and management of radiotherapy-induced fatigue. Expert Rev Qual Life Cancer Care.

[16] Rivera DR, Ganz PA, Weyrich MS, Bandos H, Melnikow J (2018). Chemotherapy-associated peripheral neuropathy in patients with early-stage breast cancer: a systematic review. J Natl Cancer Inst.

[17] Silver JK, Baima J (2013). Cancer prehabilitation: an opportunity to decrease treatment-related morbidity, increase cancer treatment options, and improve physical and psychological health outcomes. Am J Phys Med Rehabil.

[18] Heywood R, McCarthy AL, Skinner TL (2018). Efficacy of exercise interventions in patients with advanced cancer: a systematic review. Arch Phys Med Rehabil.

[19] Sweegers MG, Altenburg TM, Brug J (2019). Effects and moderators of exercise on muscle strength, muscle function and aerobic fitness in patients with cancer: a meta-analysis of individual patient data. Br J Sports Med.

[20] Nakano J, Hashizume K, Fukushima T (2018). Effects of aerobic and resistance exercises on physical symptoms in cancer patients: a meta-analysis. Integr Cancer Ther.

[21] Fukushima T, Nakano J, Hashizume K (2021). Effects of aerobic, resistance, and mixed exercises on quality of life in patients with cancer: a systematic review and meta-analysis. Complement Ther Clin Pract.

[22] Yamaguchi K, Nakanishi Y, Tangcharoensathien V (2022). Rehabilitation services and related health databases, Japan. Bull World Health Organ.

[23] (2024). Cabinet Office, Japan: Regional differences in healthcare availability [Webpage in Japanese]. https://www5.cao.go.jp/keizai-shimon/kaigi/special/reform/mieruka/tiikisa.html.

[24] (2025). Ministry of Health, Labour, and Welfare: [NDB] Homepage about the use of related information databases such as anonymous medical insurance [Website in Japanese]. https://www.mhlw.go.jp/stf/seisakunitsuite/bunya/kenkou_iryou/iryouhoken/reseputo/index.html.

[25] Mewes JC, Steuten LM, Ijzerman MJ, van Harten WH (2012). Effectiveness of multidimensional cancer survivor rehabilitation and cost-effectiveness of cancer rehabilitation in general: a systematic review. Oncologist.

[26] Iragorri N, de Oliveira C, Fitzgerald N, Essue B (2021). The out-of-pocket cost burden of cancer care—a systematic literature review. Curr Oncol.

[27] Gubler-Gut BE, Pöhlmann J, Flatz A, Schwenkglenks M, Rohrmann S (2021). Cost-effectiveness of physical activity interventions in cancer survivors of developed countries: a systematic review. J Cancer Surviv.

[28] Weaver SJ, Jacobsen PB (2018). Cancer care coordination: opportunities for healthcare delivery research. Transl Behav Med.

[29] Liang H, Tao L, Ford EW, Beydoun MA, Eid SM (2020). The patient-centered oncology care on health care utilization and cost: a systematic review and meta-analysis. Health Care Manage Rev.

